# A Review from a Clinical Perspective: Recent Advances in Biosensors for the Detection of L-Amino Acids

**DOI:** 10.3390/bios14010005

**Published:** 2023-12-22

**Authors:** Kristina Ratautė, Dalius Ratautas

**Affiliations:** 1 Faculty of Medicine, Vilnius University, M. K. Čiurlionio Str. 21, LT-03101 Vilnius, Lithuania; 2 Life Science Center, Vilnius University, Saulėtekio al. 7, LT-10257 Vilnius, Lithuania

**Keywords:** amino acids, detection, biosensors, diagnostics, clinical condition, disease

## Abstract

The field of biosensors is filled with reports and designs of various sensors, with the vast majority focusing on glucose sensing. However, in addition to glucose, there are many other important analytes that are worth investigating as well. In particular, L-amino acids appear as important diagnostic markers for a number of conditions. However, the progress in L-amino acid detection and the development of biosensors for L-amino acids are still somewhat insufficient. In recent years, the need to determine L-amino acids from clinical samples has risen. More clinical data appear to demonstrate that abnormal concentrations of L-amino acids are related to various clinical conditions such as inherited metabolic disorders, dyslipidemia, type 2 diabetes, muscle damage, etc. However, to this day, the diagnostic potential of L-amino acids is not yet fully established. Most likely, this is because of the difficulties in measuring L-amino acids, especially in human blood. In this review article, we extensively investigate the ‘overlooked’ L-amino acids. We review typical levels of amino acids present in human blood and broadly survey the importance of L-amino acids in most common conditions which can be monitored or diagnosed from changes in L-amino acids present in human blood. We also provide an overview of recent biosensors for L-amino acid monitoring and their advantages and disadvantages, with some other alternative methods for L-amino acid quantification, and finally we outline future perspectives related to the development of biosensing devices for L-amino acid monitoring.

## 1. Typical Concentrations of Total L-Amino Acids in Human Blood

Research articles indicate that human blood contains various L-amino acids. In an early article by Stein and Moore, human blood plasma was investigated and twenty-eight compounds were found to be ninhydrin-positive and identifiable as L-amino acids [1]. Quantitative analysis revealed that the L-amino acids present in human blood at the highest concentrations are glutamine, alanine, valine, proline, and lysine. The average concentration of total L-amino acids was determined to be around 37 mg per 100 mL of plasma (or ~3.3 mmol/L). However, the described study investigated a small sample size; in total, plasma from only five individuals was investigated. A more recent study by Schmidt et al. investigated the typical levels of L-amino acids in the blood of various groups based on diet preferences, i.e., meat-eaters, fish-eaters, vegetarians, and vegans. Significant sample sizes (~100) were reached [2]. The results for particular L-amino acids were different between the groups; however, the concentrations of total L-amino acids were similar in the range of 4.1–5.2 mmol/L. In a newly published paper by our group, concentrations of total L-amino acids were measured in patients undergoing renal replacement therapy. The results showed that the total L-amino acid levels in human blood were in the range 1.3–6.3 mmol/L [3]. In summary, research articles demonstrate that the total concentration of L-amino acids in human blood is significant and, for example, is comparable to the concentration of glucose. Various research studies have given different compositions of specific amino acids, most likely related to the specifics and differences of the investigated groups. However, the total level of L-amino acids in human blood is quite similar and can be in the range of 1.3 to 6.3 mmol/L. In the next section, we will review the role of L-amino acids in several conditions and thus the importance of L-amino acids for diagnostics.

## 2. Overview of L-Amino Acids in Clinical Conditions

**Figure 1:**
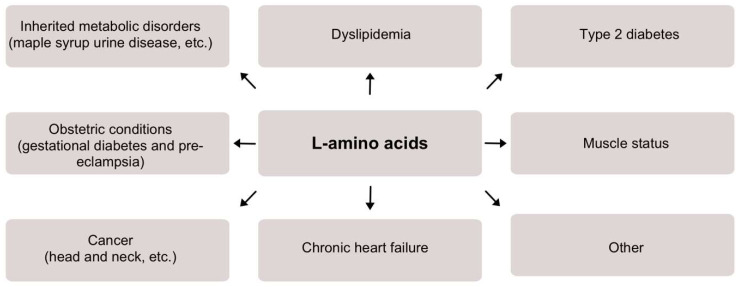
A scheme demonstrating the significance of L-amino acids detection and/or monitoring for various medical conditions.

In our opinion, the importance of L-amino acids for clinical diagnostics is not sufficiently clear and is often overlooked. Several factors are contributing to this issue, such as the relationship of L-amino acids to many biochemical processes, and, in turn, to many various conditions from metabolic disorders to cancers, giving them lower specificity. Additionally, to this date, the measurement methods and/or devices for L-amino acids are quite expensive and usually they are not routinely applied for clinical sample analysis. In this section, we focus on various clinical conditions and/or diseases related to L-amino acids and in turn highlight the importance of L-amino acids for diagnostics. The most common clinical conditions related to L-amino acids are discussed in detail and summarized in Figure 1.

### 2.1. Inherited Metabolic Disorders

Inherited metabolic disorders, such as maple syrup urine disease, are closely related to serum L-amino acids both for diagnostic and therapeutic purposes [4,5]. Maple syrup urine disease has been shown to result in an increase in branched-chain L-amino acids (BCAAs) in plasma [6]. Long-term treatment requires restriction of dietary BCAAs and supplementation with a BCAA-free mixture. Frequent monitoring of the plasma concentration of L-amino acids could help identify deficiencies in essential L-amino acids secondary to a protein-restricted diet, and thus guide treatment.

### 2.2. Dyslipidemia

BCAA, tyrosine, tryptophan, glutamate, and L-homocysteine concentrations are reported to be considerably lower in healthy diet individuals [7]. In one study, forty healthy men and women with a body mass index of 25.6 ± 0.6 were overfed by 1250 kCal per day for 28 days and, as a consequence, their serum BCAAs increased from 397 ± 10 µmol/L to 428 ± 9 µmol/L. This highlights the role of food overconsumption in increased BCAA concentrations [8]. Moreover, BCAAs and total L-amino acid levels are increased in obese individuals. Higher concentrations of all BCAAs, phenylalanine, tyrosine, and alanine are associated with a higher fat mass index, and some plasma-free L-amino acids are correlated with parameters used to measure central obesity (e.g., waist circumference, waist-to-hip ratio, BMI) [9,10]. Interestingly, medical studies report that serum amino acid profiles and increased levels of leucine, arginine, valine, proline, phenylalanine, isoleucine, and lysine could predict the development of hypertriglyceridemia later in life in healthy individuals even before they become obese [11]. For example, serum L-amino acid concentrations of 1125 individuals were analyzed with the follow-up data available after 7 years for comparison and a relationship was found between L-amino acids and an increased risk of developing hypertriglyceridemia [12]. In summary, measurements of L-amino acid concentrations can serve as early biomarkers to identify people at high risk of developing dyslipidemia and diseases related to dyslipidemia.

### 2.3. Type 2 Diabetes

Increased levels of L-amino acids have been reported to be an early manifestation of insulin resistance; i.e., changes in blood concentrations of particular L-amino acids (e.g., sulfur amino acids, tyrosine, phenylalanine) are apparent with obesity and insulin resistance, often before the onset of clinical diagnosis [13,14]. Furthermore, BCAAs and aromatic L-amino acids predict the risk of developing type 2 diabetes. For example, during a study conducted by Wang et al., 2422 normoglycemic individuals were profiled for metabolites. After 12 years, 201 individuals were diagnosed with type 2 diabetes. A significant correlation between the disease and several amino acids, i.e., isoleucine, leucine, valine, tyrosine, and phenylalanine, was established. The risk of future diabetes was concluded to be at least fourfold greater in those with high plasma amino acid concentrations in both the discovery and replication samples [15]. Similar results were obtained from the Southall And Brent REvisited (SABRE) study. Asian-American individuals have been reported to have an increased risk of diabetes compared to Europeans that is not related to obesity or established traditional metabolic measures [16]. In total, 643 Asian and 801 European non-diabetic males were tested for baseline levels of nine L-amino acids in serum. Serum concentrations of isoleucine, phenylalanine, tyrosine, and alanine were significantly higher in the Asian men, while glycemia was similar in the two groups. Diabetes developed in 35% of the Asian and 14% of the European males. Strong associations between L-amino acids and diabetes were also observed in other studies [17,18,19]. In summary, L-amino acids could serve as a predictor of early-onset type 2 diabetes in healthy individuals.

### 2.4. Obstetric Conditions

There are data showing that L-amino acids are related to some obstetric conditions, such as gestational diabetes and pre-eclampsia. Significant differences in serum levels of particular amino acids (e.g., L-arginine, L-glycine, and 3-hydroxy-isovalerylcarnitine) are observed between females who develop gestational diabetes and controls [20]. According to that article, these differences exist already in the first trimester of pregnancy and may be useful for early screening. Additionally, increased concentrations of L-amino acids were shown to be more prevalent in women with pre-eclampsia [21]. In the reviewed article, maternal and cord blood L-amino acid levels were reported to be significantly higher in women with pre-eclampsia compared to pregnant women without this condition. Most likely, pre-eclampsia is associated with increased placental L-amino acid transport or reduced utilization of fetal L-amino acids.

### 2.5. Muscle Status

A strong relationship between L-amino acids and muscle status has been reported. For example, L-amino acid imbalance could explain fatigue after long-term exercise [22]. During overtraining, brain tryptophan uptake and 5-hydroxytryptamine synthesis are increased. In a 1993 study, the serum L-amino acid profile was determined in nine athletes before and after competing in the Colmar ultratriathlon. Serum concentrations of 25 L-amino acids (22 proteinogenic L-amino acids and citrulline, α-Aminobutyric acid, and taurine) decreased by 18% with a decrease in 18 individual L-amino acids by 9–56% and an increase in tyrosine, phenylalanine, methionine, cystine, and free tryptophan. In addition, a decrease in body mass (approximately 3.3 kg) and an increase in plasma volume (approximately 7.6%) were observed. The decrease in intracellular water may be seen as a protein catabolic signal. Interestingly, it was also reported that L-amino acid supplementation could be used to prevent muscle damage. For example, during prolonged exercise, L-branched-chain amino acid (BCAA) supplementation decreases the serum concentration of intramuscular enzymes (creatine kinase and lactate dehydrogenase) and thus may reduce muscle damage [23]. L-amino acids were also reported to be important for patients who have diseases that induce muscle mass decrease. It was reported that the supplementation of BCAAs may reduce the muscle damage for patients with rheumatic diseases. It was demonstrated that plasma BCAAs, aspartic acid, and glutamate concentrations correlate positively with the rate of improvement in biceps femoris muscle atrophy, suggesting that these amino acids are associated with the BCAA-induced increase in muscle mass [24]. It was also demonstrated that, for patients treated in intensive care units, the supplementation of L-amino acids can protect skeletal muscle mass and function; for severely ill patients, a higher provision of protein and L-amino acids has been associated with a lower mortality [25]. Another recent report also indicated that significant amounts of L-amino acids are lost during continuous renal replacement therapy and may indicate the need for clinical intervention for clinical care patients [26]. It was demonstrated that the amount of L-amino acids lost during continuous renal replacement therapy is increased not only by the set-up specifics, but also by individual considerations of the patients (e.g., by a higher systemic concentration of amino acids and by a higher fat-free mass index (mostly muscles)). To summarize, L-amino acids are strongly associated with body muscle status, and they could show muscle damage and help to prevent it.

### 2.6. Cancer

L-amino acids could be an important parameter for cancer monitoring, diagnosis, and prognosis. It was reported that L-amino acids could be predictors of cancer cachexia and sarcopenia [27]. Cancer cachexia and sarcopenia cause ongoing muscle loss and a higher serum essential/total L-amino acid ratio is associated with sarcopenia in patients with advanced gastrointestinal cancers. It is reported that the levels of L-amino acids are also associated with the stage of disease and prognosis in patients with head and neck cancer [28]. Increased levels of alpha-aminobutyric acid, aminoadipic acid, L-histidine, L-proline, and L-tryptophan are associated with a reduced risk of advanced stage head and neck cancer. Increased levels of beta-alanine, beta-aminobutyric acid, ethanolamine, glycine, isoleucine, 4-hydroxyproline, and phenylalanine are associated with an increased risk of advanced stage head and neck cancer. Increased levels of alpha-aminobutyric acid are associated with increased overall survival, while increased levels of arginine, ethanolamine, glycine, histidine, isoleucine, 4-hydroxyproline, leucine, lysine, 3-methylhistidine, phenylalanine, and serine are associated with decreased overall survival. Other papers also report a relationship between cancers and L-amino acids. For example, in a recent review by Kang, the importance of L-amino acid restriction for cancer therapy was highlighted [29]. It was stated that L-amino acid restriction, in particular L-leucine, could be a simple metabolic intervention for cancer therapy.

### 2.7. Chronic Heart Failure

Plasma L-amino acids can predict chronic heart failure. It was demonstrated that 17 L-amino acids and two concentration ratios between specific L-amino acids were significantly different in a heart failure group compared with those in the control [30]. The heart failure group had five specific L-amino acids, i.e., L-monoethanolamine, L-methionine, L-tyrosine, L-methylhistidine, and L-histidine, in correlation with cardiac function indicators such as B-type natriuretic peptide, left ventricle ejection fraction, and others. Moreover, in another study, it was shown that concentrations of essential L-amino acids and L-BCAAs are significantly lower for patients with severe chronic heart failure, which is associated with low nutritional status along with the loss of skeletal muscle [31]. To summarize, research papers indicate that chronic heart failure is related to changes in L-amino acid concentrations.

### 2.8. Other

A relationship between L-amino acids and other conditions also were reported. Several papers have highlighted the importance of L-amino acids, especially L-BCAAs, for the prediction of septic shock resolution and survival [32,33]. A significant number of papers report that L-amino acids play important roles in brain functioning and could be related to various mental and neurological conditions, such as autism spectrum disorders, cerebral palsy, and Parkinson’s disease [34,35,36,37]. Papers have been published indicating a relationship between L-amino acids and liver cirrhosis [38,39] and chronic kidney disease [40]. Some papers also reported that L-amino acids may be related to a patient’s frailty and longevity in general [41,42]. For example, Hamed et al. reported that L-amino acids could be important in age-related diseases and that protein and/or L-amino acid restriction could be related with reduced occurrence in cancer, diabetes, and overall mortality [42]. Another paper also indicated that L-amino acids are highly important in aging [43].

## 3. Biosensors for Measurement of Total L-Amino Acid Concentration

### 3.1. General Overview of Enzymatic Biosensors

Biosensors are devices incorporating biological elements of recognition, i.e., enzymes, DNA and/or aptamers for the detection of specific analytes [44]. Most common biosensors are enzymatic; enzymes, mostly of the oxidoreductase class, are immobilized on the surface of an electrode. Enzymatic biosensors have several advantages in comparison to other sensors: usually they have better sensitivity and specificity, they can be made portable, they can be cost-effective for a single measurement, and they can be miniaturized and applied for point-of-care diagnostics [45]. Typically, enzymes used to design biosensors conduct catalytic reactions and, as a result, produce a substrate (e.g., H_2_O_2_, NH_4_^+^, H_3_O^+^) which can be detected using an electrochemical approach and related with the concentration of the initial analyte [46]. For example, the first biosensors, proposed by Clark and Lyons in 1962, were enzymatic and suited for glucose detection. These biosensors were based on an enzymatic membrane containing glucose oxidase (GOx), a reference electrode, and a sensing electrode (e.g., pH, pO_2_) for glucose detection [47]. In the proposed design, the GOx layer oxidized glucose to form gluconic acid, which caused a drop in pH depending on the concentration of glucose. The change in pH could be measured using an electrochemical approach applying potentiometry. Perhaps the first biosensor constructed and demonstrated to operate in biological samples (human blood and serum) for glucose measurement was created by Updike and Hicks and reported in an article “The Enzyme Electrode” in *Nature* [48]. In their work, glucose oxidase was used together with acrylamide gel to form a membrane of 25–50 µm thickness. The membrane was placed on the surface of a platinum electrode catalyzing the reduction of oxygen. Once glucose was introduced, the membrane started to oxidize glucose and, as a result, reduce the concentration of oxygen. The oxygen concentration decrease was monitored using a platinum electrode and was inversely proportional to the concentration of glucose: the greater the concentration of glucose, the lower the platinum electrode current that was observed.

**Figure 2:**
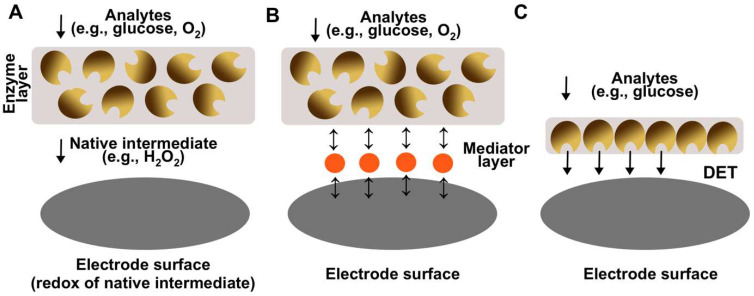
A scheme demonstrating different generations of enzymatic biosensors. (**A**) The first-generation biosensors are based on electron transfer to the electrode via native intermediate of enzymatic reaction (e.g., H_2_O_2_). (**B**) The second-generation biosensors are based on electron transfer to the electrode via a redox active intermediate. (**C**) The third-generation biosensors are based on direct electron transfer between the enzyme and the electrode.

The work of Updike and Hicks provided many insights and general guidelines for the design of biosensor electrodes. Firstly, it was demonstrated that the enzymatic membrane (or enzymatic layer in general) should have a significant excess of enzyme and be relatively thick. The excess and thickness are required to ensure the stability of the biosensor by ensuring that the electrode operates in the mass transfer process as a rate-determining step [49]. In this way, the loss in enzymatic activity does not significantly influence the signal of a biosensor. Secondly, it was shown that the reaction of interest (e.g., glucose oxidation) should be the limiting one. For example, the response of a platinum electrode towards oxygen should be independent with respect to the solution flow rate and oxygen flux, and only dependent on the activity of glucose oxidase. Finally, it was proposed that the second electrode with the inactivated enzyme could be utilized to measure the interference resulting from components present in a complex sample. The designed electrode was the first-generation of biosensors since the electron transfer was facilitated via native redox intermediates of the enzyme (O_2_ and H_2_O_2_) (Figure 2A). Apart from glucose, first-generation biosensors were developed for the detection of L-glutamate [50,51], ethanol [52], L-lactate [53], L-ascorbic acid [54], cholesterol [55], choline [56], and various phenols [57]. Despite their simple and convenient properties, first-generation biosensors have flaws, since the electron transfer is being conducted via a native mediator (usually H_2_O_2_) and typically is detected by amperometric (anodic) measurement at relatively high potential. This approach has several limitations: firstly, endogenous reducing compounds such as uric and ascorbic acids and some common medicines (e.g., acetaminophen) can also be oxidized at high potential [58]; secondly, changes in oxygen flux can influence and distort the result of biosensors’ measurements. As a result, the selectivity of such sensors may suffer since unknown compounds could also contribute to the signal and thus make the measurements less accurate. 

As a possible solution, second-generation biosensors were introduced which incorporated artificial redox-active mediators typically immobilized on the electrode surface. Electron transfer occurs between the enzyme and the mediator instead of an oxygen/hydrogen peroxide couple (MET biosensors, Figure 2B) [59]. The key advantage of using artificial mediators could be the significant decrease in a redox potential and, as a result, the decrease in interference resulting from the oxidation of compounds present in complex matrixes. Various electron transfer mediators were proposed, such as ferrocene [60], quinones [61], tetrathiofulvalene [62], metal redox complexes (e.g., osmium-complex) [63], and conducting polymers (e.g., polyaniline) [64]. The usage of those redox mediators allowed for better biosensing systems operating at lower electrochemical potentials and avoiding electrochemical interference. In some cases, the usage of redox-active mediators was necessary to create a biosensor, since the enzymes used in biosensor design had native electron acceptors poorly suited for electrochemical detection (e.g., irreversible oxidation) [62]. Many biosensors which could be attributed to the second generation were designed for the detection of glucose [65], glycerol [62], and other such analytes [66]. However, despite significant improvements, MET biosensors have certain shortcomings. The most significant one is the loss of mediator from the electrode surface and in turn the decrease in activity of the biosensor over time. For example, previously we designed an MET biosensor for glycerol detection using the redox-active mediator tetrathiafulvalene (TTF). Despite very low solubility, the mediator still managed to dissolve into solution and “escape” from the electrode surface, which resulted in a contribution to the decrease in electrode activity [62]. This shortcoming is even more apparent when higher solubility mediators are being used. 

For the above discussed reasons, the third-generation biosensors were introduced operating via the direct electron transfer principle (DET biosensors, Figure 2C) [67]. For DET biosensors, a mediator is not needed since electron transfer is facilitated from the enzyme active center to the electrode directly. DET biosensors offer several advantages in comparison to previous generations: lower operational potential and thus lower interference, greater current outputs and thus greater sensitivity, and lower limits of detection. Many electrodes applicable as third-generation biosensors have been demonstrated for the oxidation of various compounds [68,69,70,71,72,73,74,75]. For example, our group recently designed a DET bioanode applicable as a glucose biosensor based on DET-capable glucose dehydrogenase (GDH) [69]. The electrode had an unprecedented sensitivity of 0.715 A M^−1^ cm^−2^, it could measure glucose in the range of 5.0–25.0 µM (1000-fold lower compared to the relevant clinical range), and the glucose addition to signal time was only a few seconds. However, despite exceptional performance, DET biosensors have significant disadvantages. Firstly, DET is difficult to achieve since the active center of the enzyme should be suitable for the DET, i.e., close to the enzyme surface, and thus the number of enzymes which can operate via DET is limited. Secondly, DET biosensors have low operational stability due to a limited number of enzymes operating on the surface. Typically, only the first monolayer can participate in the DET [76]; thus, the biosensor activity decrease cannot be compensated by an excess of enzyme molecules, and enzyme activity decrease directly lowers the activity of the biosensor. This is especially apparent when analyzing real complex samples where matrix medium has a negative effect on enzymatic activity. For example, in our work, the DET electrode activity dropped to only around 10% (in comparison to initial activity) at constant operation in human blood for 24 h [69]. Advantages and disadvantages of all generations of enzymatic electrodes are summarized in Table 1. 

### 3.2. Overview of Biosensors for L-Amino Acid Detection

Several biosensors have been reported for L-amino acid measurements in various matrixes, and some were tested using real clinical samples. One of the first biosensors for L-amino acid detection was published in 1974 by Nanjo and Guilbault in an article named “Enzyme electrode for L-amino acids and glucose” [77]. Biosensors were constructed for glucose and L-amino acids based on glucose oxidase and L-amino acid oxidase, respectively. Measurements were carried out by observing the current change resulting from the reduction of the dissolved oxygen in a solution at a negative potential (−0.6 V vs. SCE), where sensitivity was found to be greater in comparison to measurements at positive potential (+0.6 V vs. SCE) when observing oxidation of hydrogen peroxide. The biosensor for L-amino acids was tested using various L-amino acids including L-methionine, L-leucine, L-phenylalanine, L-tryptophan, L-proline, L-serine, and others. As expected, it was demonstrated that L-amino acid oxidase does not catalyze the oxidative deamination of tested L-amino acids at equal rates. For example, the biosensor had a greatest activity towards L-methionine and L-leucine, while L-proline and L-serine did not react at all. As a result, it was postulated that the biosensor could be suitable for the detection of certain L-amino acids for specific applications. The biosensor was stable for 4 months with little loss of activity; however, no clinically or industrially relevant samples were reported to have been measured using the designed biosensor.

Further attempts to create L-amino acid biosensors were industry-focused. For example, Varadi et al. developed a bienzymatic system containing both L- and D-amino acid oxidases to stereospecifically measure amino acid enantiomers during the brewing process [78]. A system proposed in a report was made of thin-layer enzymatic cells with L- and D-amino acid oxidase, immobilized covalently on a protein membrane. This biosensor was able to determine both L- and D-amino acids separately by measuring hydrogen peroxide produced by the enzyme. As expected, L/D-amino acid oxidase did not oxidize the investigated amino acids at similar rates. It was demonstrated that enzyme was most active towards tryptophan, leucine, and methionine, while serine and threonine activity was insignificant. A similar system, containing L- and D-amino acid oxidases, was introduced by Sarkar et al. [79]. Enzymes were immobilized on a screen-printed rhodinized carbon working electrode and applied to monitor milk aging effects. It is worth noting that, to this point, all the discussed systems were first-generation biosensors.

A second-generation biosensor for detecting L-amino acids was demonstrated by Lata and Pundir [80]. In this study, goat kidney L-amino acid oxidase was immobilized on a carboxylated multiwalled carbon nanotubes/nickel hexacyanoferrate/polypyrrole hybrid film. That work represents one of the earliest instances of utilizing nanomaterials (specifically, multiwalled carbon nanotubes, MWCNT) to enhance biosensor parameters. The designed hybrid film acted as a mediator, ensuring electrode performance at a reduced electrochemical potential (0.15 V vs. Ag/AgCl). The biosensor’s efficacy was assessed for detecting L-amino acids in fruit juices and various alcoholic beverages, and tested for interferences caused by common compounds known to affect biosensor signals (e.g., ascorbic acid).

**Figure 3:**
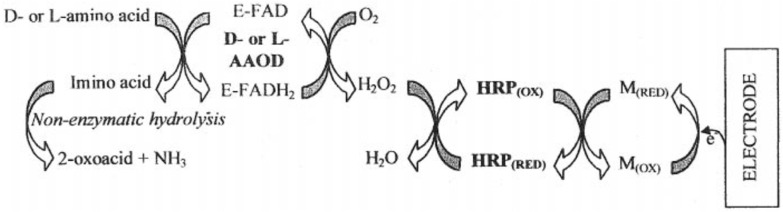
A schematic diagram displaying the enzyme electrode design for the determination of L- and D-amino acids. E-FAD/E-FADH_2_—D/L amino acid oxidase (reduced/oxidized), HRP—horseradish peroxidase, M—mediator (ferrocene). Reprinted from Dominguez et al. [81] with permission from Elsevier.

More complex systems were developed, for example, involving several enzymes for the oxidation of L-amino acids. Dominguez et al. proposed a second-generation biosensor system, where L- or D-amino acid oxidase, together with horseradish peroxidase and mediator ferrocene, were immobilized in the bulk of a graphite electrode (Figure 3) [81]. The key difference and advantage of the Dominguez et al. report was that the biosensor operated at significantly reduced electrochemical potential, around 0.00 V vs. Ag/AgCl, thus avoiding possible electrochemical interference. As in previous works, significant differences were observed in enzymatic activity of the biosensor towards different amino acids. For example, tryptophan had the highest activity, while serine almost did not react at all. The developed biosensor was used to measure indicatory values of amino acids in grapes for wine-making quality control with good success.

**Figure 4:**
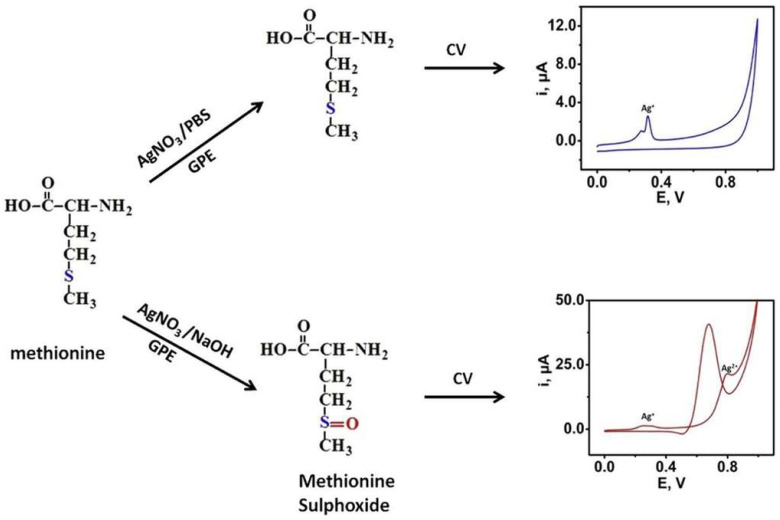
A schematic diagram demonstrating an enzyme-free sensor for D/L-methionine detection in human serum samples based on AgO modified graphite electrode. Reprinted from Odewunmi et al. [82] with permission from Elsevier.

The determination of L-amino acids in clinical samples is a complex task and only a few sensors have been reported. However, all of them were developed for the analysis of the specific L-amino acid, rather than total. Odewunmi et al. demonstrated a sensor for D/L-methionine detection in human serum samples based on AgO micro/nanoparticle-modified graphite electrode (Figure 4) [82]. However, the detection in serum was based on standard addition, i.e., methionine was added to serum sample and afterwards analyzed. Additionally, serum samples required pretreatment, i.e., proteins were removed prior to measurements.

Another sensor was developed by Garcia-Carmona et al. for detecting L-tyrosine in plasma and whole blood samples [83]. The sensor was fabricated using filtered multi-walled carbon nanotubes through a Teflon filter. The utilization of nanomaterials allowed the development of an enzyme-free sensor capable of selectively measuring L-tyrosine and demonstrating adequate analytical parameters (see Table 2). The analysis of L-tyrosine was conducted using differential pulse voltammetry, revealing significant differences in L-tyrosine levels between healthy samples and those from tyrosinemia patients.

**Figure 5:**
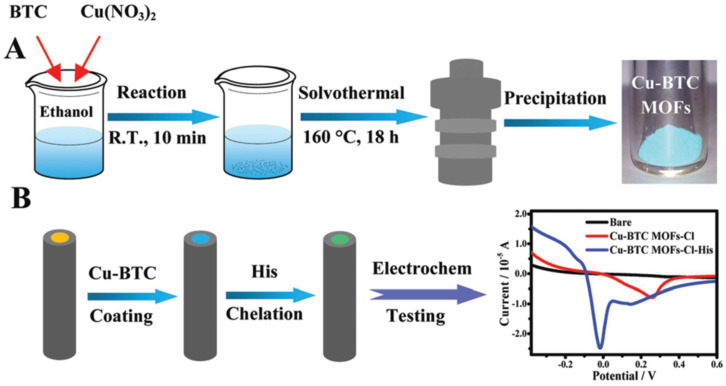
A schematic diagram displaying (**A**) the preparation of Cu organic framework and (**B**) electrode modification procedure and electrochemical Cu redox before/after exposure to histidine. Reprinted from Hua et al. [84] with the permission from Royal Society of Chemistry.

One of the most innovative recent advancements in histidine detection was demonstrated by Hua et al. (Figure 5) [84]. In that study, tetrahedral copper metal–organic frameworks were immobilized on the electrode surface and utilized to chelate histidine. In our opinion, this work represents one of the most elegant applications of nanomaterials in sensing. The copper metal–organic frameworks ensured the selectivity of the sensing system, rendering a biological recognition element unnecessary—the designed sensor was enzyme-free. Using voltammetry, it was observed that Cu redox was diminished in the presence of Cl^−^ in the medium due to the formation of CuCl_2_. However, upon the introduction of histidine into the sample, a Cu-His complex formed, increasing the Cu redox process. Furthermore, the modified electrode was successfully employed in detecting added histidine in blood samples.

**Figure 6:**
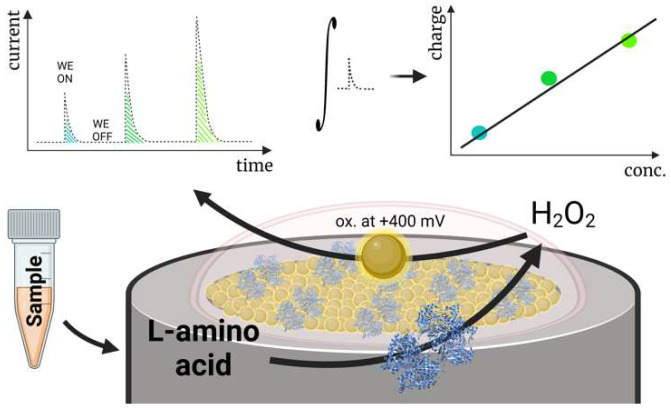
A schematic diagram displaying a capacitance-based biosensor for the detection of total L-amino acids operating in human blood serum. Reprinted from Miškinis et al. [3] according to Creative Commons license (CC-BY 4.0.).

Our group recently developed a biosensor for detecting total L-amino acids applicable in clinical diagnostics (Figure 6) [3]. The biosensor was created by immobilizing gold nanoparticles (AuNP) on a Pt electrode and covering the electrode with a membrane containing crosslinked L-amino acid oxidase. We investigated the biosensor using a novel capacitance-based principle: measuring electrode capacitance after electrode polarization, disconnecting it from the circuit, and adding the respective amount of analyte. The capacitance-based measurements aimed to allow the enzymatic membrane to oxidize most L-amino acids diffusing to the surface, storing the electrons received from oxidized H_2_O_2_ on the electrode surface. The function of the nanomaterials (AuNP) in the design was to ensure charge accumulation on the electrode surface. Experiments without AuNP showed that electrodes had significantly reduced capacitance and were hardly applicable using this method to detect L-amino acids. Consequently, the received capacitance during the measurement was mostly influenced by the total concentration of L-amino acids, despite the activity difference of the enzyme L-amino acid oxidase for various L-amino acids. The designed biosensor was used to assess the relative loss of L-amino acids in patients undergoing renal replacement therapy by measuring L-amino acid levels in human serum samples before and after entering/leaving the hemodialysis apparatus.

In addition to biosensors for L-amino acid detection, several other methods and/or approaches could be also reviewed which can be applicable for the measurement of L-amino acids from clinical samples. One of the most commonly available alternatives to measure L-amino acids is commercially available colorimetric L-amino acid measurement kits (e.g., Sigma-Aldrich’s (St Louis, MI, USA) kit for L-amino acid quantification, catalog number: MAK002). Compared to biosensors, L-amino acid kits have advantages and disadvantages. Advantages include availability, the high number of samples that can be analyzed at the same time in parallel, and typically lower LOD compared to biosensors. To add more, R&D-only L-amino acid measurement kits can be validated and adjusted to be applied in clinical laboratories for high throughput screening for L-amino acids. A significant reduction in price can be achieved in cases where the entire kit is used fully, i.e., the maximum number of samples is analyzed from a single kit. Common disadvantages of L-amino acid kits are the price per measurement (much greater in comparison to biosensors) and the requirement to pretreat samples prior to the measurements (remove proteins, filter, etc.). In our opinion, colorimetric kits are especially useful as an alternative method for L-amino acid measurements during biosensor R&D work.

Another attractive alternative for measuring L-amino acids is commercially available home testing services for L-amino acid quantification. For example, Cerascreen GmbH offers a commercially available amino acid test which measures in total 15 specific L-amino acids. The test is based on dried blood spot analysis; Cerascreen GmbH sends a specific kit with the instructions for how to place a blood sample on a special card. Once fully dried, the sample is shipped to the laboratory where the amino acids are analyzed from the prepared dried blood sample. At this time, home testing services offering L-amino acid analysis are the most affordable and most available way to quantify most amino acids.

### 3.3. Current Challenges Limiting the Applicability of L-Amino Acid Biosensors for Clinical Applications and Possible Solutions

Many papers have been published describing biosensors for the measurement of total and/or specific amino acids [85]. However, only a minority of them are dedicated to measuring exclusively total L-amino acids. Some of the sensors were tested by measuring food industry samples (e.g., brewing or fruit juice), although it should be noted that appropriate evaluation using biological clinical samples is still lacking, possibly limiting the speed of adoption of L-amino acid biosensors for clinical applications. Several shortcomings exist in the detection principles and in understanding the need to detect L-amino acids. Their solution could enhance the development of biosensors for the detection of L-amino acids and their application. Those disadvantages could be separated into medical issues and technological challenges.

First of all, one of the most apparent shortcomings is not technological but rather medical—the lack of a clear clinical understanding regarding the value of total L-amino acid measurements. As discussed in the clinical section of this article, L-amino acids are associated with numerous disorders and diseases. However, as diagnostic markers, they might lack specificity. Detecting and predicting diseases may require a specific profile of L-amino acids to be determined [86]. Given the difficulty in measuring L-amino acids, establishing a stronger relationship could indeed be challenging. One potential solution could involve more frequent or even routine measurement of amino acid concentrations whenever feasible. This approach could accumulate more data on L-amino acid concentrations in human blood, potentially establishing stronger correlations with various clinical conditions.

The second major challenge related to detecting L-amino acids is more technological in nature. We believe the primary difficulty in determining the concentration of total main L-amino acids lies in the need to ascertain the concentration of numerous individual L-amino acids with varying reactivities. For instance, in the creation of enzyme-based biosensors for L-amino acid determination, L-amino acid oxidase is commonly employed. This enzyme can oxidize nearly all L-amino acids, albeit at different rates [87]. L-leucine and L-arginine are generally noted as the best substrates for L-amino acid oxidase, while L-glutamic and L-aspartic acids are considered the least reactive amino acids [88]. Consequently, biosensor performance can significantly differ when applied to samples with similar total L-amino acid concentrations but different individual compositions. To address this technological challenge, various approaches could be considered. One potential approach involves designing a bioreactor-type biosensor capable of oxidizing all L-amino acids within a fixed volume, thereby minimizing the influence of differing activities on the sensor’s final outcome. For example, Wang et al. demonstrated an elegant reactor-type biosensor for biochemical oxygen demand (BOD) measurements [89]. In their work, a reactor utilizing immobilized microbial cell beads as a recognition bio-element was developed. These beads were freely suspended (not immobilized) in the aqueous solution, enabling better BOD measurements compared to typical membrane-based biosensors. Alternatively, the challenge could be addressed by employing a better optimized and more finely tuned method during the operation of L-amino acid oxidase. For instance, in our research, we designed a biosensor for total L-amino acid measurement using the capacitance-based principle instead of amperometry. This method allowed even the less active L-amino acids to contribute to the signal, yielding results comparable to alternative measurement methods [3].

## 4. Concluding Remarks and Future Perspectives of L-Amino Acid Detection

In our opinion, at the moment, the importance of L-amino acid detection is mostly overlooked, and there is significant room for improvement. Scientific data demonstrate that total L-amino acids are associated with many conditions and diseases. Strong relationships have been demonstrated between L-amino acids and inherited metabolic disorders (e.g., maple syrup disease), dyslipidemia, type 2 diabetes, and muscle damage. Reports also give evidence to connect the detection of L-amino acids with other conditions such as pre-eclampsia, cancers, and cardiovascular diseases. A significant amount of clinical data indicates that L-amino acids could be an important parameter for the monitoring and diagnosis of these conditions. This summary of articles related to L-amino acid detection also indicates that scientific work has been conducted to design biosensors suitable for the detection and quantification of total and specific L-amino acids. However, the number of sensors reported is not great. Although the published papers were mostly based on the use of the relatively universal enzyme L-amino acid oxidase for the detection, it could not be unnoticed that the detection of L-amino acid still has significant technological challenges. Most of them involve difficulties in measuring all the amino acids, since they have different substrate specificities toward L-amino acid oxidases.

We believe that the detection and monitoring of L-amino acids is a developing field of biosensing since the need to determine L-amino acids from a clinical perspective is solid. Novel biosensing technologies will be developed which could solve the technological problems mentioned above, resulting in more comprehensive data sets about the levels of amino acids in the human body in the presence (or absence) of various medical conditions. In this way, the clear clinical need will push forward technological developments, while new biosensing technologies will, in turn, contribute to more inclusive clinical data, strengthening each other. As a result, we envision that L-amino acid testing will be a routine test in many clinical facilities in the near future and even more technologies (such as L-amino acids testing from dried blood spots) will be developed, making L-amino acid testing widely available to the public and contributing to the general well-being of society.

## Figures and Tables

**Table 1:** Key advantages and disadvantages of enzymatic biosensors and their generations.

Generation	Key Advantages	Key Disadvantages
First (electrons are being transferred via native mediator, e.g., H_2_O_2_)	Simple to design Native mediator is constantly being produced by the enzyme, thus cannot be depleted The sensing layer can be separated from the electrode surface The sensing layer can be made using excessive amount of enzyme to thus increase stability	Depended on the concentration of the dissolved oxygen in a solution Lack of sensitivity High operational potential (e.g., +0.6 V vs. Ag/AgCl)
Second (electrons are being transferred via synthetic mediator, e.g., tetrathiafulvalene)	Usually do not depend on the concentration of the dissolved oxygen in a solution Have better sensitivities in comparisonto first generation The sensing layer can be made using excessive amount of enzyme to thus increase stability Low operational potential (e.g., +0.2 V vs. Ag/AgCl)	Complex design Synthetic mediator needs to be constantly added to the solution Desorption of mediator from the surfacecan occur
Third (electrons are being transferred directly)	Usually do not depend on the concentration of the dissolved oxygen in a solution Highest sensitivities in comparison to the first and second generations Lowest operational potentials (e.g., 0 V vs. Ag/AgCl) No mediator (native or synthetic) is needed for the electrode operation	Most complex design Can be implemented for a limited number of selective enzymes Low operational stability in comparison to other generations

**Table 2:** A comparative table of most significant biosensors for L-amino acid detection (majority for total L-amino acids, some for specific).

Biosensor	Enzyme, Nanomaterials Used	Analysis Method	Analytical Parameters (as Reported)	Real Sample
Nanjo and Guilbault [77]	Enzyme: L-amino acid oxidase (*Crotalus adamanteus*) Nanomaterials: N/A	Chronoamperometry	Sensitivity: N/A LOD: 10^−6^–10^−5^ M Linear range: N/A Stability: stable for at least 4 months	N/A
Varadi et al. [78]	Enzyme: L-amino acid oxidase (*Crotalus adamanteus*), D-amino acid oxidase (porcine kidney) Nanomaterials: N/A	Flow-through amperometry	Sensitivity: N/A LOD: N/A Linear range: 0.1–3 mM Stability: 900–1000 measurements	Brewing process samples (ginger and brown beer)
Sarkar et al. [79]	Enzyme: L-amino acid oxidase (*Crotalus adamanteus*), D-amino acid oxidase (porcine kidney) Nanomaterials: N/A	Chronoamperometry	Sensitivity: N/A LOD: 0.15–0.47 mM Linear range: 0.47–2.5 mM Stability: 40% activity loss after 56 days	Milk, fruit juice, urine
Lata and Pundir [80]	Enzyme: L-amino acid oxidase (goat kidney) Nanomaterials: MWCNT	Linear square voltammetry	Sensitivity: N/A LOD: 0.5 µM Linear range: 0.5 µM–100 mM Stability: 70% left after 140 days	Fruit juices, alcoholic beverages
Dominguez et al. [81]	Enzyme: L-amino acid oxidase (*Gratelis Adamate*) *, D-amino acid oxidase (porcine kidney) Nanomaterials: N/A	Chronoamperometry	Sensitivity: 10–480 µA M^−1^ LOD: 1.1–160 µM Linear range: 10^−6^–10^−4^ M Stability: around 10 days with no need to regenerate the electrode surface	Grapes
Odewunmi et al. [82]	Enzyme: enzyme-free Nanomaterials: AgO nanoparticles	Chronoamperometry	Sensitivity: 4230 µA mM^−1^ cm^−2^ LOD: 0.42 µM Linear range: 60–500 µM Stability: N/A	Human serum
Garcia-Carmona et al. [83]	Enzyme: enzyme-free Nanomaterials: MWCNT	Differential pulse voltammograms	Sensitivity: 0.012 µA µM^−1^ LOD: 8 µM Linear range: 25–750 µM Stability: N/A	Human serum
Hua et al. [84]	Enzyme: enzyme-free Nanomaterials: tetrahedral copper metal–organic frameworks	Linear sweep voltammetry	Sensitivity: N/A LOD: 25 nM Linear range: N/A Stability: no significant change after 6-month storage	Human blood
Miškinis et al. [3]	Enzyme: L-amino acid oxidase (*Crotalus adamanteus*) Nanomaterials: gold nanoparticles	Chronocoulometry	Sensitivity: 0.73 µC/µM LOD: 5.5 µM Linear range: 5.5–100 µM Stability: 50% of the initial activity after 10 days of storage	Human serum and blood

* As reported in the article.
